# Rheuma-VOR – gesundheitsökonomische Bewertung einer frühzeitigen rheumatologischen Diagnostik: Zusammenfassung des G-BA-Berichtes

**DOI:** 10.1007/s00393-026-01858-6

**Published:** 2026-09-18

**Authors:** Juliana Rachel Hoeper, Matthias Dreher, Julian Maximilian Kohl, Marlon Grodd, Johanna Callhoff, Andreas Schwarting, Jan Zeidler

**Affiliations:** 1 Center for Health Economics Research Hannover (CHERH), Leibniz Universität Hannover, Königsworther Platz 1, 30167 Hannover, Deutschland; 2 Schwerpunkt Rheumatologie und klinische Immunologie, Universitätsmedizin Mainz, Langenbeckstraße 1, 55131 Mainz, Deutschland; 3 Programmbereich Epidemiologie, Deutsches Rheuma-Forschungszentrum Berlin, Berlin, Deutschland; 4 Institut für Medizinische Biometrie und Statistik, Universitätsklinikum Freiburg, Freiburg, Deutschland; 5 Koordinierungszentrum für Klinische Studien (KKS), Medizinische Fakultät, Universitätsklinikum, Heidelberg, Deutschland; 6 RZ Rheumazentrum Rheinland-Pfalz GmbH, Bad Kreuznach, Deutschland

**Keywords:** Rheumatische Erkrankungen, Rheuma-VOR, Kosten-Nutzwert-Analyse, Direkte und indirekte Gesundheitskosten, Frühzeitige Diagnostik, Rheumatic diseases, Rheuma-VOR, Cost-effectiveness analysis, Direct and indirect healthcare costs, Early diagnosis

## Abstract

**Hintergrund:**

Rheuma-VOR wurde initiiert, um lange Wartezeiten bis zur fachärztlichen Diagnosestellung und leitliniengerechte Therapieeinleitung bei rheumatoider Arthritis (RA), Psoriasisarthritis (PsA) und axialen Spondyloarthritiden (axSpA) zu verkürzen.

**Methodik:**

Rheuma-VOR ist eine prospektive, nichtrandomisierte, multizentrische Netzwerkstudie, in die eine gesundheitsökonomische Evaluation eingebettet wurde. Mithilfe eines quasi-experimentellen Kohortendesigns und Propensity-Score-Matching wurde die Interventionsgruppe (Rheuma-VOR) mit der Standardversorgung (Kerndokumentation) verglichen. Analysiert wurden direkte Kosten (Medikamente, Krankenhaus- und Rehabilitationsaufenthalte) sowie indirekte Kosten (Produktivitätsverluste) aus gesellschaftlicher Perspektive.

**Ergebnisse:**

Rheuma-VOR erzielte Gesamteinsparungen von 3,84 Mio. € gegenüber der Standardversorgung, vor allem durch weniger Krankenhausaufenthalte (−1,15 Mio. €), reduzierte Arzneimittelausgaben (−2,03 Mio. €) und verbesserte Arbeitsfähigkeit (−0,70 Mio. € indirekte Kosten). Weniger Biologika waren zum Indexzeitpunkt verordnet (2,4 % vs. 11,8 %). Das Modell zeigte ein dominantes Kosten-Effektivitäts-Profil: vergleichbare oder bessere gesundheitsbezogene Lebensqualität bei geringeren Gesamtkosten (Rheuma-VOR: 2.445,17 €/Patient, 95 % KI 1.955,79–3.041,50 €; Standardversorgung: 3.107,33 €/Patient, 95 % KI 1.958,28–3.997,62 €). Die jährlichen Koordinationsstellenkosten (272,35 €/Patient) wurden durch Einsparungen mehr als ausgeglichen.

**Diskussion:**

Strukturierte, sektorenübergreifende Versorgungsmodelle wie Rheuma-VOR reduzieren diagnostische Verzögerungen, nutzen Ressourcen effizienter und sind ökonomisch tragfähig. Langfristig sind weitere Einsparungen durch Vermeidung irreversibler Schäden zu erwarten.

**Zusatzmaterial online:**

Die Online-Version dieses Beitrags (https://doi.org/10.1007/s00393-026-01858-6) enthält Anhang 1 bis 7.

## Abstract (en)

**Background:**

Rheuma-VOR was initiated to reduce long waiting times for specialist diagnosis and guideline-based therapy initiation in rheumatoid arthritis (RA), psoriatic arthritis (PsA), and axial spondyloarthritis (axSpA).

**Methods:**

Rheuma-VOR is a prospective, nonrandomized, multicenter network study with an embedded health economic evaluation. Using a quasi-experimental cohort design and propensity score matching, the intervention group (Rheuma-VOR) was compared with standard care (national database). Direct costs (medications, hospital, and rehabilitation) and indirect costs (productivity losses) were analyzed from a societal perspective.

**Results:**

Rheuma-VOR achieved total savings of € 3.84 million versus standard care, mainly due to fewer hospitalizations (−€ 1.15 million), reduced drug costs (−€ 2.03 million), and improved work capacity (−€ 0.70 million indirect costs). Fewer biologics were prescribed (2.4 % vs. 11.8 %). The model demonstrated a dominant cost-effectiveness profile: similar or better health-related quality of life at lower total costs (Rheuma-VOR: € 2,445.17/patient, 95 % confidence interval [CI] € 1,955.79–€ 3,041.50; standard care: € 3,107.33/patient, 95% CI € 1,958.28–€ 3,997.62). Annual coordination center costs (€ 272.35/patient) were fully offset by savings.

**Conclusion:**

Structured, cross-sectoral care models like Rheuma-VOR reduce diagnostic delays, optimize resource use, and are economically sustainable. Long-term benefits include additional savings through prevention of irreversible damage.

Entzündlich-rheumatische Erkrankungen wie die rheumatoide Arthritis, Psoriasisarthritis und axiale Spondyloarthritis verursachen erhebliche direkte und indirekte Kosten. Verzögerte Diagnosen führen zu höherer Krankheitslast, vermehrten Krankenhausaufenthalten, Produktivitätsverlusten und kostenintensiven Therapien. Eine frühzeitige Diagnosestellung und Therapieeinleitung könnte Krankheitsverläufe verbessern und gleichzeitig gesundheitsökonomische Ressourcen effizienter nutzen.

## Hintergrund und Fragestellung

Entzündlich-rheumatische Erkrankungen wie rheumatoide Arthritis (RA), Psoriasisarthritis (PsA) und axiale Spondyloarthritiden (axSpA) betreffen in Deutschland rund 1,8 Mio. Menschen und verursachen hohe Krankheitslasten sowie erhebliche sozioökonomische Kosten [1,2]. Trotz medikamentöser Fortschritte bestehen weiterhin Versorgungslücken, die den Zugang zur fachärztlichen Betreuung verzögern und Krankheitsverläufe ungünstig beeinflussen [2,3].

Eine zentrale Herausforderung ist die verzögerte Diagnosestellung. Die Leitlinien der European League Against Rheumatism (EULAR) empfehlen innerhalb von 3 Monaten nach Symptombeginn eine krankheitsmodifizierende Therapie (DMARDs) einzuleiten [4]. In der Versorgungspraxis berichten jedoch 25 % der RA- und 58 % der axSpA-Patient:innen über Symptombeginn > 1 Jahr vor Diagnose; mediane Zeiten betragen 4 Monate (RA) bzw. 18 Monate (axSpA) [5]. Diese Verzögerungen verhindern nicht nur den rechtzeitigen Therapiebeginn, sondern führen auch zu einer erhöhten Krankheitsaktivität, einer schlechteren Prognose und zu irreversiblen strukturellen Schäden an Gelenken und Organen [3].

Die Ursachen dieser Verzögerungen sind vielschichtig. Der Mangel an internistischen Rheumatologen führt zu Terminengpässen und langen Wartezeiten. Dies stellt in der Versorgung ebenso Hindernisse dar wie die oft fehlende gezielte Zuweisung durch die Primärversorgung.

Der demografische Wandel verschärft diese Situation: ein erheblicher Anteil der derzeit tätigen Rheumatolog:innen steht kurz vor dem Renteneintritt, während gleichzeitig die Prävalenz chronischer Erkrankungen in der alternden Bevölkerung weiter ansteigt. Viele Patient:innen – 32 % bei RA und 54 % bei axSpA – sind gar nicht rheumatologisch angebunden. Diese Betroffenen werden seltener leitliniengerecht behandelt und erhalten ihre Diagnose im Mittel deutlich später (2 Jahre bei RA, 5,7 Jahre bei axSpA) [2].

Die ökonomischen Konsequenzen dieser Versorgungslücken sind signifikant. Bereits im Jahr 2006 beliefen sich die direkten Kosten entzündlich-rheumatischer Erkrankungen in Deutschland auf 3,80 Mrd. €, wovon 1,46 Mrd. € auf die Versorgung entzündlicher Polyarthropathien (ICD-10 M05–M14) entfielen. Bis zum Jahr 2020 hatten sich die direkten Versorgungskosten für diese Krankheitsgruppe auf 3,06 Mrd. € mehr als verdoppelt. Der größte Anteil mit über 55 % entfiel auf Arzneimittelkosten, insbesondere Biologika, deren Kosten jedoch zuletzt durch die Einführung von Biosimilars gesenkt werden konnten. Die ambulante (20 %) und stationäre Versorgung (12 %) bilden nicht nur weitere Kostenpositionen, sondern stehen zugleich in engem Zusammenhang mit erheblichen indirekten Kosten, etwa infolge krankheitsbedingter Arbeitsausfälle und Erwerbsunfähigkeiten. Sie stellen nach wie vor die zentralen indirekten Kostenfaktoren dar, auch wenn durch eine verbesserte Versorgung der Anteil der Patient:innen mit niedriger Krankheitsaktivität von 35 % auf 46 %, sowie die Erwerbstätigenquote von 39 % auf 53 % in den letzten Jahren gesteigert werden konnte. Frühzeitige Diagnosestellung und kontinuierliche Behandlung sind daher entscheidend für die Kosteneffizienz, während Therapieunterbrechungen mit Mehrkosten verbunden sind [2].

Die gesundheitsökonomische Bewertung entzündlich-rheumatischer Erkrankungen erfolgt auf Basis von Ressourcenverbrauch und patientenrelevanten Outcomes. Neben direkten Kosten (Arztbesuche, Medikamente, Hospitalisationen) spielen insbesondere indirekte Kosten durch Produktivitätsverluste eine zentrale Rolle [6]. Chronische Erkrankungen verursachen aufgrund ihrer Progression erhebliche Kosten, während eine frühzeitige und leitliniengerechte Behandlung sowohl Ausgaben senken als auch die gesundheitsbezogene Lebensqualität erhalten kann [7]. Für RA wurde gezeigt, dass Arzneimittelkosten bis zu 87 % der direkten Kosten ausmachen, während indirekte Kosten bis zu 86 % der Gesamtkosten verursachen können; ähnliche Muster zeigen sich bei PsA und axSpA [8–10]. Diese Kostenstruktur unterstreicht die Bedeutung von Frühdiagnoseprogrammen, die eine Senkung des Bedarfs an Biologika ermöglichen könnten.

Vor diesem Hintergrund wurden innovative Versorgungsprojekte wie ADAPTHERA und Rheuma-VOR etabliert. Rheuma-VOR nutzt ein sektorenübergreifendes Netzwerk mit zentraler Koordination, strukturierter Triage und schneller Terminvermittlung [11,12]. Im Zuge von Rheuma-VOR wurden 7.049 Verdachtsdiagnosen an die Koordinationszentren gemeldet, wovon 1.537 Diagnosen gestellt wurden [11]. Die durchschnittliche Zeit vom Auftreten der Symptome bis zur Diagnose betrug bei rheumatoider Arthritis 0,55 Jahre gegenüber 2,31 Jahren in der Kerndokumentation, bei Psoriasisarthritis 2,43 gegenüber 4,41 Jahren und bei axialer Spondyloarthritis 3,92 gegenüber 8,41 Jahren. Im Mittel betrug die Wartezeit vom Eingang der Verdachtsdiagnose bis zum Termin 30 ± 37 Tage. Zusätzlich ist in Rheinland-Pfalz eine durchschnittliche Wartezeit von 16 ± 15 Tagen zu berücksichtigen, die durch den 15-minütigen Screening-Termin vor der fachärztlichen Vorstellung entsteht [11].

Diese frühzeitige Diagnostik führte nicht nur zu einer Verbesserung der gesundheitlichen Outcomes, sondern zeigte auch Potenzial für gesundheitsökonomische Einsparungen, wie bereits in der Rheuma-VOR Hauptpublikation dargelegt [11]. Langfristig sind zusätzliche Einsparungen plausibel, da durch die Prävention irreversibler Schäden und den Erhalt der Arbeitsfähigkeit Folgekosten reduziert werden könnten [13]. Ergänzt wurde Rheuma-VOR durch mobile Screening-Formate wie die Rheuma-Bus-Tour, die insbesondere ländliche Regionen erreichten [14,15].

Eine frühzeitige Diagnosestellung und Therapieeinleitung ermöglichen es, Krankheitsprogression und irreversible Schäden zu verhindern [3]. Dadurch können langfristig nicht nur die individuellen Krankheitslasten reduziert, sondern auch erhebliche finanzielle Ressourcen eingespart werden [16]. Die konsequente Nutzung dieser Potenziale eröffnet die Chance, bestehende Versorgungsdefizite auszugleichen, die vorhandenen Mittel effizienter einzusetzen und zusätzliche Investitionen in die rheumatologische Versorgung zu ermöglichen.

## Studiendesign und Untersuchungsmethoden

Rheuma-VOR ist eine prospektive, multizentrische Proof-of-Concept-Studie in Deutschland, mit dem Ziel, diagnostische Verzögerungen bei Patient:innen mit entzündlich-rheumatischen Erkrankungen zu reduzieren. Primäres Studienziel war die Verkürzung der Zeitspanne zwischen Symptombeginn, rheumatologischer Vorstellung und gesicherter Diagnosestellung.

Hierzu wurde mit 72 rheumatologischen Zentren und Schwerpunktpraxen in 4 Bundesländern (Berlin, Niedersachsen, Rheinland-Pfalz und Saarland) ein standardisiertes strukturiertes Triage- und Zuweisungskonzept implementiert. Dieses basiert auf erkrankungsbildspezifischen an Klassifikationskriterien adaptierte Screeningfragebogen, definierten Patientenpfaden sowie optimierten Überweisungsalgorithmen zur verbesserten Identifikation bzw. Stratifizierung potenziell entzündlich-rheumatischer Erkrankungen, um die rheumatologischen Kapazitäten zu entlasten [11,17].

Das Studiendesign zielte auf eine Beschleunigung der diagnostischen Abklärung unter gleichzeitiger Sicherstellung einer leitlinienkonformen Versorgung ab. Während die Effektevaluation, die vorwiegende klinische und Patient-Reported-Outcome-Parameter beinhaltet, Patient:innen vom 1. Juli 2017 bis zum 31. Dezember 2020 mit Basis- und 12-Monats-Follow-up-Daten berücksichtigt, stützt sich die gesundheitsökonomische Evaluation auf eine Analyse von Primär- und Registerdaten, die in diesem Zeitraum erhoben wurden [11]. Das Studiendesign und die übersichtsgebenden Daten wurden bereits publiziert [11].

Die Evaluation konzentrierte sich auf den Vergleich von direkten und indirekten Kosten zwischen den Versorgungsmodellen aus gesellschaftlicher Perspektive unter Verwendung von Daten aus bis zu zwei klinischen Visiten pro Patient:in, abhängig von der Datenverfügbarkeit. Angesichts des Real-World-Settings und des Fehlens einer Randomisierung bot der gematchte Kohortenansatz einen pragmatischen Rahmen zur Beurteilung ökonomischer Ergebnisse und Effektivität.

Die vorgestellten Ergebnisse sind Bestandteil des Berichtswesens gegenüber dem G‑BA im Zuge der Evaluation von Rheuma-VOR und stellen eine Zusammenfassung der zentralen gesundheitsökonomischen Resultate dar.

### Datenaufbereitung und Matching

Alle Daten wurden in einem anonymisierten Format an das Center for Health Economics Research Hannover (CHERH) übermittelt. Unter Verwendung eines quasi-experimentellen gematchten Kohortendesigns vergleicht die Studie Patient:innen, die im Rahmen der Rheuma-VOR-Studie eingeschlossen wurden (Interventionsgruppe, Rheuma-VOR), mit Patient:innen, die eine Standardversorgung erhielten (Kontrollgruppe, Kerndokumentation der regionalen kooperativen Rheumazentren) [18]. Da die Gruppenzuweisung nicht randomisiert, sondern auf bestehenden Versorgungsstrukturen basierte, wurde ein Propensity-Score-Matching unter Verwendung von Alter (mit einer Toleranz von ± 5 Jahren), Geschlecht, Hauptdiagnose und Anzahl der rheumatologischen Visiten durchgeführt, um die Vergleichbarkeit sicherzustellen und um den Selektionsbias zu minimieren. Aufgrund der erreichten Matching-Qualität waren keine weiteren Adjustierungen erforderlich.

Vor dem Matching enthielt der Rheuma-VOR-Datensatz 945 Patient:innen mit Daten ausschließlich für Visite 1 sowie 592 mit Daten für sowohl Visite 1 als auch Visite 2. Der Datensatz der Kerndokumentation umfasste entsprechend 937 und 1.195 Patient:innen.

Zur Ermöglichung robuster longitudinaler Analysen wurde der gematchte Datensatz in zwei analytisch getrennte Gruppen unterteilt.

Es wurden 806 Patient:innen in die Analysegruppe „nur Visite 1“ eingeschlossen (Gesamt *n* = 1.612). Diese Fälle repräsentieren Patient:innen, für die keine Follow-up-Daten vorlagen, meist aufgrund des Endes des Studienzeitraums und nicht wegen Drop-outs.

Die zweite Gruppe bestand aus 589 Patient:innen pro Gruppe mit vollständigen Daten für sowohl Visite 1 als auch Visite 2 (Gesamt *n* = 1.178).

Insgesamt wurden drei analytische Teilsets definiert:

- Gesamtheit der Visite-1-Stichprobe,
- Teilset mit ausschließlich Visite-1-Daten,
- Teilset mit Daten für beide Visiten.

Diese Differenzierungen waren entscheidend, um Kostendifferenzen eindeutig zeitabhängigen Veränderungen in Behandlung oder Gesundheitsstatus zuschreiben zu können. Die Werte dieser Subgruppen wurden in Tabellenform dargestellt, um Übersichtlichkeit und Vergleichbarkeit sicherzustellen.

Fallverluste traten in der Kerndokumentation stärker auf, mit 131 nicht gematchten Fällen im Teilset „nur Visite 1“ und 606 im Teilset „Visite 1 und 2“, im Vergleich zu 139 bzw. 3 Fällen im Rheuma-VOR-Datensatz. Da die Rheuma-VOR-Kohorte die Interventionsgruppe darstellte und die Berechnung der Gesundheitskosten primär auf der Erfassung von Veränderungen über die Zeit – insbesondere zwischen den Visiten – basierte, wurde die Reduktion der Stichprobengröße als akzeptabel erachtet.

**Abb. 1:**
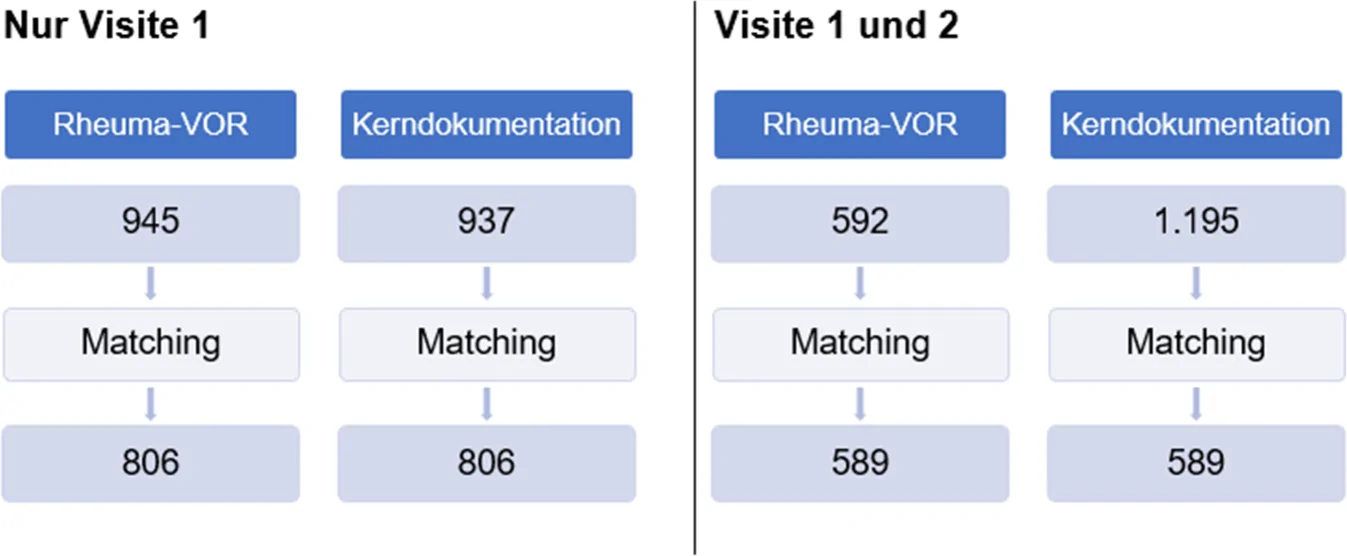
Veränderungen der Anzahl von Studienteilnehmenden durch das Matching

Eine Übersicht über die Veränderungen der Anzahl von Studienteilnehmenden durch das Matching ist in Abb. 1 ersichtlich.

Die genutzten Angaben zum „Erhebungsjahr“ und „Erhebungsmonat“ der Kerndokumentation beziehen sich auf das Datum des ersten Kontakts mit einem Rheumatologen und nicht zwangsläufig auf den Zeitpunkt der tatsächlichen Datenerhebung. Aus diesem Grund kann der Zeitraum zwischen der Visite und der ersten Verschreibung eines Medikaments lediglich approximativ berechnet werden. Das Propensity-Score-Matching dient als Anpassungstechnik zur Kontrolle von Confounding und gleicht zentrale Charakteristika zwischen den Kohorten aus.

### Umgang mit fehlenden Daten

Fehlende Daten wurden durch vordefinierte Annahmen behandelt, anstatt mittels Listwise-Deletion oder Imputation. Diese umfassten Ableitungsregeln, die auf Abhängigkeiten zwischen Variablen und klinischer Plausibilität basierten. So wurden beispielsweise fehlende Enddaten von Medikationen als fortlaufende Behandlung interpretiert, sofern keine widersprüchlichen Daten vorlagen. Aufgrund der Struktur der Daten wurde eine multiple Imputation als ungeeignet erachtet.

### Kostenberechnungen der Outcomes

Die Haupt-Outcomes umfassten sowohl direkte als auch indirekte Gesundheitskosten sowie patientenzentrierte Messgrößen. Direkte Kosten beinhalteten Medikamentenausgaben, Krankenhausaufenthalte und Rehabilitationsleistungen.

Indirekte Kosten wurden anhand von Krankheitstagen erfasst, wodurch Schätzungen von Produktivitätsverlusten nach dem Friction-Cost-Ansatz ermöglicht wurden.

Die gesundheitsbezogene Lebensqualität wurde ebenfalls als zentraler Outcome betrachtet und ermöglichte die Berechnung von Kosten-Nutzen-Quotienten wie dem inkrementellen Kosten-Effektivitäts-Verhältnis (ICER). Für Patient:innen mit Daten aus beiden Visiten wurden Veränderungen der Lebensqualität über die Zeit genutzt, um die Kosten zu schätzen, die erforderlich sind, um eine Verbesserung um 0,1 Punkte (10 %) zu erzielen.

Abschließend wurden die Implementierungskosten auf Basis der jährlichen Personalkosten je Patient errechnet.

#### Medikamentenkosten

Die Aufbereitung der Medikationsdaten erforderte Annahmen bei unvollständigen oder inkonsistenten Datumsangaben.

Fehlende „End-Daten“ führten zur Annahme einer fortlaufenden Therapie, sofern keine gegenteiligen Hinweise vorlagen. „End-Daten“ waren nur für ausgewählte Medikamente wie Methotrexat oral und parenteral (MTX), Leflunomid, Sulfasalazin verfügbar, nicht jedoch für Biologika. Umgekehrt wurden Einträge, die ein „Start-Datum“ ohne zugehörige Medikamenten- oder Dosierungsangaben enthielten, nicht als valide Verordnungen gewertet. Wo hingegen nur eine Dosierung, aber kein Medikamentenname dokumentiert war, wurde angenommen, dass eine gültige Verordnung vorlag, und das Medikament wurde entsprechend als verschrieben codiert. Diese Vorgehensweise ermöglichte eine konsistente Auswertung der Verordnungsfrequenzen.

Für jedes Medikament wurde die größte verfügbare Packungsgröße (N3) priorisiert; wenn nur N1- oder N2-Größen verfügbar waren, wurden die Kosten proportional angepasst.

Medikamentenkosten wurden während des Beobachtungszeitraums des Rheuma-VOR-Projekts aus der Lauer-Taxe (Lauer-Taxe 4.0) abgeleitet, wobei Preisdaten quartalsweise (jeweils zum 15. Februar, Mai, August und November) veröffentlicht wurden [19]. Für diesen Zeitraum wurden durchschnittliche Medikamentenpreise berechnet. Nur für Therapien mit erheblichen Preisschwankungen wurden individuelle Quartalspreise angewendet, um deren Variabilität genauer abzubilden.

Die Arzneimittelkosten wurden in verschiedene Kategorien aufgeteilt: Gesamtausgaben für Pharmaka, Biologika-Therapien, csDMARDs und Glukokortikoide. Diese Kategorisierung ermöglichte eine detailliertere ökonomische Bewertung, insbesondere angesichts der unterschiedlichen Kostenstrukturen, Behandlungsprotokolle und Kostenvariabilität in den einzelnen Gruppen. Biologika-Therapien wurden aufgrund ihrer überproportional hohen Kosten separat analysiert, während Basistherapien unter einer einheitlichen Variablen zusammengefasst wurden. Glukokortikoide wurden einzeln betrachtet, da sie eine erhebliche Variabilität in Dosierung und Applikationsmustern aufwiesen. Um eine differenzierte Bewertung longitudinaler Verschreibungstrends zu ermöglichen, wurde eine Variable erstellt, die angibt, ob eine bestimmte Therapie ausschließlich bei Visite 1, ausschließlich bei Visite 2 oder bei beiden Zeitpunkten verschrieben wurde.

Aufgrund fehlender Unterscheidung zwischen Originator-Biologika und Biosimilars basierten Kostenberechnungen auf Originator-Preisen, was potenziell zu Überschätzungen führen könnte. Dateninkonsistenzen wurden durch Plausibilitätsprüfungen und Standardisierung adressiert.

Aufgrund fehlender Datumsangaben war es nicht immer möglich zu bestimmen, ob die dokumentierte Verschreibung den initialen rheumatologischen Kontakt markierte oder ob Biologika bereits zuvor verabreicht worden waren.

Folglich wurden keine Aufsättigungsdosen in die Berechnung der Behandlungskosten einbezogen. Stattdessen wurde, in Anlehnung an literaturbasierte Empfehlungen, eine durchschnittliche Behandlungsdauer von 9 Monaten angenommen, wenn retrospektive 12-Monats-Daten lediglich angaben, ob ein Biologikum zu irgendeinem Zeitpunkt in diesem Zeitraum verabreicht wurde [20,21].

#### Krankenhausaufenthalte

Krankenhauskosten wurden berechnet, indem die Anzahl der Krankenhausaufenthalte in jeder Kohorte mit der jeweiligen durchschnittlichen Verweildauer in den vorangegangenen 12 Monaten multipliziert wurde. Dieses Produkt wurde anschließend mit einem standardisierten Tagessatz multipliziert, um die Gesamtkosten für stationäre Behandlungen zu ermitteln. Historische Trends bei Krankenhauskosten wurden durch eigene Berechnungen auf Basis von Huscher et al. ermittelt [16]. Diese Veränderungen wurden mithilfe des Verbraucherpreisindex kontextualisiert, der im Jahr 2011 einen Wert von 100 %, 2015 für die Kategorie „Gesundheit“ 92,2 % und 2019 105,3 % auswies [22]. Nach Inflationsanpassungen über diese Referenzjahre hinweg wurde der standardisierte Tagessatz für 2019 auf 78,08 € festgelegt. Obwohl diese Zahl eine konsistente Grundlage für die Kostenberechnung bietet, kann nicht ausgeschlossen werden, dass bestimmte Änderungen in der zugrundeliegenden Kostenstruktur im Zeitverlauf nicht vollständig abgebildet wurden, was zu leichten Abweichungen von den tatsächlichen historischen Tagessätzen führen könnte.

#### Rehabilitationsleistungen

Rehabilitationskosten wurden analog berechnet, indem die Anzahl der Rehabilitationsaufenthalte innerhalb der vorangegangenen 12 Monate mit der durchschnittlichen Aufenthaltsdauer in Wochen und anschließend mit dem standardisierten Tagessatz für Rehabilitationsleistungen multipliziert wurde, um die Gesamtausgaben zu ermitteln.

#### Produktivitätsverluste

Bei der gesundheitsökonomischen Bewertung von Produktivitätsverlusten können sowohl der Human-Capital- als auch der Friction-Cost-Ansatz angewendet werden. Den vorliegenden Berechnungen liegt durchgehend der Friction-Cost-Ansatz zugrunde. Er geht von einem Friktionszeitraum von 90 Tagen aus und passt den Produktivitätsverlust auf 80 % der vollen Arbeitskosten an, um kurzfristige Teilersatzmöglichkeiten der Arbeitskraft zu berücksichtigen [23]. Aufgrund des Fehlens einkommensspezifischer Daten in der Kohorte wurden nationale Durchschnittswerte der Arbeitskosten verwendet. Die Grundlage bildeten Krankheitstage in den 12 Monaten vor jedem Erhebungszeitpunkt.

Die Berechnungen der Produktivitätsverluste wurden auf den Daten aus dem Jahr 2019 standardisiert. Im 2019 arbeiteten Arbeitnehmer:innen im Durchschnitt 1.383 h an 207 effektiven von 251 potentiellen Arbeitstagen [24]. Unter Verwendung der durchschnittlichen Arbeitskosten pro Stunde für 2019 (35,90 €) wurden die jährlichen Arbeitskosten pro Arbeitnehmer:in auf 49.649,70 € geschätzt, was 197,81 € pro Arbeitstag und 136,03 € pro Kalendertag entspricht. Friktionskorrigierte Äquivalente wurden mit 158,25 € bzw. 109,04 € berechnet [25]. Zum Vergleich berichteten Huscher et al. Krankheitskosten pro Tag von 96,06 € im Jahr 2002 und 102,56 € im Jahr 2011, was den hier ermittelten Schätzungen nahekommt [16].

Zur Quantifizierung arbeitsbezogener Produktivitätsverluste wurde der durchschnittliche Arbeitskostenwert pro Kalender- oder Arbeitstag mit der Anzahl der Krankheitstage multipliziert. Diese Berechnungen berücksichtigten Krankheitstage in den 12 Monaten vor der Datenerhebung, während andauernde Krankheitszeiten zum Zeitpunkt der Erhebung separat berechnet wurden. Durchschnittliche Krankheitsdauern wurden als standardisierte Schätzwerte beibehalten, um Konsistenz in den Kostenberechnungen über retrospektive und aktuelle Angaben hinweg sicherzustellen. Dieser Ansatz ermöglichte eine einheitliche und robuste Quantifizierung indirekter Produktivitätsverluste.

Ebenso wurde die vorzeitige Berentung aufgrund der rheumatischen Erkrankungen berücksichtigt.

#### Implementierungskosten

Schließlich wurden die Betriebskosten der Rheuma-VOR-Koordinationsstellen auf Basis des Personalbedarfs und der Gehälter berechnet, einschließlich mindestens einer Rheumatolog:in in Teilzeit (etwa ein Drittel bis ein halbes Vollzeitäquivalent, abhängig vom Bundesland), einer rheumatologischen Fachassistenz (RFA) in Vollzeit und einer Sekretariatskraft in Vollzeit. Für konservative Berechnungen wurde das Personal auf Vollzeitäquivalente standardisiert: 107.608,80 € für Rheumatolog:innen, 46.207,20 € für RFA und 45.177,60 € für Sekretariatskräfte, was insgesamt 198.993,60 € jährlich pro Zentrum ergibt.

Diese Infrastrukturkosten wurden den erzielten Einsparungen z. B. aufgrund der Verschiebung des Therapiestarts bei Medikamenten, Krankenhausaufenthalten und Produktivität gegenübergestellt, um den Nettonutzen der Intervention zu bewerten.

Gemäß den Empfehlungen für gesundheitsökonomische Evaluationen wurden Inflationsanpassungen vorgenommen, um Werte aus früheren Jahren und zukunftsgerichtete Projektionen zu berücksichtigen. Hierfür wird die Anwendung des gesamtwirtschaftlichen Preisindex empfohlen. Internationale Leitlinien schlagen häufig die Anwendung eines Diskontierungssatzes von 3 % oder 5 % vor [26]. Zur Sicherstellung der Vergleichbarkeit über die Zeit hinweg wurden alle Geldbeträge unter Verwendung des Verbraucherpreisindex angepasst, wobei das Jahr 2015 als Basisjahr (100 %) diente. Sensitivitätsanalysen wurden durchgeführt, um die Kostenvariabilität unter verschiedenen Inflations- und Diskontierungsszenarien zu bewerten.

### Statistische Analyse

Die statistischen Analysen wurden mit IBM SPSS Statistics (Version 26) und R (Version 4.0.3) durchgeführt, ergänzt durch die R‑Pakete *haven* (Version 2.4.3), *readxl* (Version 1.3.1), *tibble* (Version 3.1.5), *data.table* (Version 1.14.2), *purrr *(Version 0.3.4), *dplyr* (Version 1.0.7), *magrittr* (Version 2.0.1) und *reshape2* (Version 1.4.4).

Der Chi-Quadrat-Test wurde angewendet, um Zusammenhänge zwischen nominalen oder ordinalen kategorialen Variablen – wie etwa dem Vorhandensein oder Nichtvorhandensein eines Krankenhausaufenthaltes – zu prüfen.

Für Gruppenvergleiche hinsichtlich ordinaler Variablen, wie Kosten und Dauer des Krankenhausaufenthaltes, wurde der nichtparametrische Mann-Whitney-U-Test eingesetzt.

## Ergebnisse

Die gesundheitsökonomische Evaluation zeigte Gesamteinsparungen durch Rheuma-VOR gegenüber der Regelversorgung über alle Ressourcenbereiche hinweg, die im Folgenden vorgestellt werden.

### Medikamentenkosten

#### csDMARDs

Gemäß den Leitlinien zum Management der frühen rheumatoiden Arthritis wurden zu Visite 1 in beiden Kohorten vergleichbare Basistherapien eingesetzt [3]. Methotrexat war dabei das am häufigsten verordnete csDMARD (Rheuma-VOR 54,8 % vs. Kerndokumentation 48,9 %). In der gematchten Kohorte der Kerndokumentation erfolgte signifikant häufiger eine orale MTX-(*p* < 0,001) sowie Sulfasalazin-Verordnung (*p* < 0,001), während parenterales MTX in der Rheuma-VOR-Kohorte häufiger verschrieben wurde (*p* < 0,001). Zu Visite 2 lagen die gesamten Verordnungen der csDMARDs in der Kerndokumentation höher (Tab. 1; für weitere Details siehe Anhang 1 bis 4 im elektronischen Zusatzmaterial online).

#### Glukokortikoide

Aufgrund der initial höheren Krankheitsaktivität wurden in Rheuma-VOR zu Studienbeginn signifikant häufiger Glukokortikoide verordnet (*p* = 0,029). Im weiteren Verlauf konnte deren Einsatz jedoch deutlich reduziert werden, sodass zu Visite 2 signifikant weniger Patient:innen in Rheuma-VOR Glukokortikoide erhielten als in der Kerndokumentation (20,7 % vs. 43,0 %; *p* < 0,001). Bemerkenswert ist zudem, dass zu Visite 2 ein größerer Anteil der Rheuma-VOR-Kohorte ohne jegliche medikamentöse Therapie war (27,7 % vs. 17,0 %) (Tab. 1; für weitere Details siehe Anhang 1 bis 4 im elektronischen Zusatzmaterial online).

#### Biologika

Die Unterschiede zwischen den Kohorten zeigten sich auch beim Einsatz von Biologika. Zu Visite 1 erhielten lediglich 2,4 % der Rheuma-VOR-Patient:innen, jedoch 11,8 % der Kerndokumentation eine Biologika-Therapie. In Visite 2 glichen sich die Verordnungsraten zwischen beiden Gruppen an (14,6 % vs. 16,0 %; *p* = 0,743). Über den gesamten Studienzeitraum hinweg wurde bei 112 Patient:innen in Rheuma-VOR und 203 Patient:innen in der Kohorte der Kerndokumentation erstmals ein Biologikum verordnet.

Die mittleren Medikamentenkosten für Patient:innen, die irgendeine Medikation erhielten, betrugen 678,19 € (SD 2.144,23 €; *n* = 1.050) in Rheuma-VOR und 2.018,60 € (SD 5.039,83 €; *n* = 1.023) in der Kerndokumentation.

Zu Visite 2 erhielten 14,6 % der Patient:innen in der Rheuma-VOR-Kohorte und 16,0 % in der Kerndokumentation eine Biologika-Therapie. Gleichzeitig waren in Rheuma-VOR deutlich mehr Personen ohne jegliche Medikation (27,7 % vs. 17,0 %).

Die mittleren Arzneimittelkosten unter den behandelten Patient:innen bei Visite 2 betrugen 2.284,61 € (SD 4.851,30 €; *n* = 426) in Rheuma-VOR und 2.561,43 € (SD 5.402,33 €; *n* = 489) bei der Kerndokumentation.

In der Rheuma-VOR-Kohorte zeigten 87 Patient:innen (14,8 %) zwischen Visite 1 und 2 eine Veränderung in der Biologika-Therapie. Bei 74 Personen wurde eine Biologika-Therapie neu initiiert, während bei 12 Patient:innen ein zuvor verordnetes Biologikum nicht mehr dokumentiert war, ohne dass ein Wechsel erfolgte; in 6 dieser Fälle lag ein dokumentierter Absetzgrund vor. Für die übrigen 6 Patient:innen war ein tatsächliches Absetzen nicht eindeutig zu rekonstruieren. Bei einer weiteren Person wurde ein Wechsel der Biologika-Therapie dokumentiert.

In der Kohorte der Kerndokumentation wiesen 74 Patient:innen (12,6 %) Therapieänderungen auf. In 61 Fällen wurde eine Biologika-Therapie neu begonnen, bei 10 Patient:innen beendet (davon 2 mit dokumentiertem Absetzgrund), und in 3 Fällen erfolgte ein Therapiewechsel. Sechs Patient:innen hatten zu beiden Zeitpunkten dasselbe Biologikum dokumentiert, jedoch gleichzeitig einen Absetzgrund, sodass der Verlauf der Medikation nicht eindeutig bestimmbar war (Tab. 1; für weitere Details siehe Anhang 1 bis 4 im elektronischen Zusatzmaterial online).

**Tab. 1:** Gesundheitsökonomische Aspekte bei Rheuma-VOR und der Kerndokumentation

	Rheuma-VOR	Kerndokumentation	Signifikanz
Anzahl Biologika *n* (%) V1	34 (2,4)	165 (11,8)	*p* < 0,001
Anzahl Biologika *n* (%) V2	86 (14,6)	94 (16,0)	*p* = 0,743
Krankenhausaufenthalte *n* (%) V1	101 (7,2)	219 (15,7)	*p* < 0,001
Krankenhausaufenthalte *n* (%) V2	55 (9,3)	75 (12,7)	*p* = 0,002
Krankenhausaufenthalte MW (SD; Median) V1	14,32 (29,64; 10)	14,04 (18,60; 10)	*p* = 0,085
Krankenhausaufenthalte MW (SD; Median) V2	13,98 (7,57; 14)	11,31 (6,69; 10)	*p* = 0,019
Rehabilitationsleistungen *n* (%) V1	73 (5,2)	120 (8,6)	*p* < 0,001
Rehabilitationsleistungen *n* (%) V2	90 (15,2)	65 (11,0)	*p* < 0,001
Rehabilitationsleistungen	4,20 (2,76; 3)	3,44 (1,35; 3)	*p* = 0,288
Anzahl Tage Reha MW (SD; Median) V1			
Rehabilitationsleistungen	4,56 (4,40; 3)	3,45 (1,89; 3)	*p* = 0,027
Anzahl Tage Reha MW (SD; Median) V2			
Produktivitätsverluste	215 (15,4)	155 (11,1)	*p* = 0,954
Derzeit AU *n* (%) V1			
Produktivitätsverluste	36 (6,1)	55 (9,3)	*p* < 0,001
Derzeit AU *n* (%) V2			
Produktivitätsverluste	367 (26,3)	337 (24,2)	*p* < 0,001
Letzte 12 Monate AU *n* (%) V1			
Produktivitätsverluste	130 (22,1)	122 (20,7)	*p* < 0,001
Letzte 12 Monate AU *n* (%) V2			
Produktivitätsverluste	38,15 (64,96; 15)	50,08 (77,15; 21)	*p* = 0,023
Anzahl Tage MW (SD; Median) V1			
Produktivitätsverluste	78,48 (123,39; 20)	84,68 (117,02; 24)	*p* = 0,446
Anzahl Tage MW (SD; Median) V2			

Prozentangaben zu Visite 1 bezogen auf *n* = 1.395 je Kohorte, zu Visite 2 auf *n* = 589 je Kohorte.

### Krankenhausaufenthalte

Die Verkürzung der Wartezeit bis zur Diagnosestellung war mit einer geringeren Rate an Krankenhausaufenthalten assoziiert. In der Rheuma-VOR-Kohorte berichteten zur Visite 1 signifikant weniger Patient:innen, in den vergangenen 12 Monaten aufgrund ihrer rheumatischen Erkrankung stationär behandelt worden zu sein, als in der Kerndokumentation (7,2 % vs. 15,7 %; *p* < 0,001). Die mediane Aufenthaltsdauer unterschied sich zwischen den Gruppen nicht (10 Tage in beiden Kohorten; *p* = 0,085).

Auch zur Visite 2 blieb dieser Unterschied bestehen: 9,3 % der Rheuma-VOR-Teilnehmenden versus 12,7 % der Kerndokumentations-Kohorte berichteten von einem stationären Aufenthalt (*p* < 0,002). Hier zeigte sich jedoch eine signifikant kürzere Aufenthaltsdauer in der Kerndokumentation (Median 10 Tage; *p* = 0,019). Insgesamt führte dies zu Einsparungen von 1.099.816,62 € bei Visite 1 und 53.663,61 € bei Visite 2 (Tab. 1; für weitere Details siehe Anhang 5; Tab. 2).

### Rehabilitationsleistungen

Zur Visite 1 nahmen in der Rheuma-VOR-Kohorte signifikant weniger Patient:innen eine Rehabilitation in Anspruch als in der Kerndokumentation (5,2 % vs. 8,6 %; *p* < 0,001). Die Dauer der stationären Rehabilitation unterschied sich nicht zwischen den Gruppen (Median 3 Wochen; *p* = 0,288).

Im weiteren Verlauf stieg der Anteil rehabilitativer Maßnahmen in der Rheuma-VOR-Kohorte auf 15,2 % (Visite 2) an. Damit berichteten zu Visite 2 signifikant mehr Patient:innen der Rheuma-VOR-Kohorte von einer Rehabilitation als in der Kohorte Kerndokumentation (11,0 %; *p* < 0,001). Dies könnte darin begründet sein, dass die frühzeitige Diagnose die Voraussetzung für die Stellung einer Reha-Indikation darstellt.

Die Rehabilitationskosten verringerten sich um 58.044,64 € bei Visite 1, während die erhöhte Inanspruchnahme bei Visite 2 zu zusätzlichen Kosten von 101.742,13 € führte, die möglicherweise durch die verbesserte Arbeitsfähigkeit kompensiert wurden (Tab. 1; für weitere Details siehe Anhang 6; Tab. 2).

### Produktivitätsverluste

Zur Visite 1 unterschieden sich die Anteile der aktuell krankgeschriebenen Patient:innen zwischen den Kohorten nicht (15,4 % Rheuma-VOR vs. 11,1 % Kerndokumentation; *p* = 0,954). Innerhalb der zurückliegenden 12 Monate lagen die Krankschreibungen jedoch signifikant häufiger in der Rheuma-VOR-Kohorte vor (*p* < 0,001), während deren Dauer in der Kerndokumentation länger war (38,2 vs. 50,1 Tage; *p* = 0,023).

Zur Visite 2 zeigte sich ein gegenläufiges Bild: Die Rate aktueller Krankschreibungen war in Rheuma-VOR niedriger (6,1 % vs. 9,3 %; *p* < 0,001), die Häufigkeit von Krankschreibungen innerhalb der letzten 12 Monate dagegen höher (22,1 % vs. 20,7 %; *p* < 0,001). Unterschiede in der Dauer bestanden nicht (*p* = 0,446). Betrachtet man ausschließlich Erwerbstätige und Auszubildende, glich sich die Häufigkeit krankheitsbedingter Arbeitsausfälle zwischen den Kohorten an (*p* = 0,370).

Bezüglich vorzeitiger Berentung berichteten 4 Patient:innen der Rheuma-VOR-Kohorte bereits zu Studienbeginn über einen krankheitsbedingten Renteneintritt, im Verlauf insgesamt 5. In der gematchten Kohorte der Kerndokumentation lagen zu Beginn 20 entsprechende Angaben vor, bei Visite 2 weitere 13. Verbesserungen der Arbeitsfähigkeit erzielten zusätzliche Einsparungen von 385.506,50 € bei Visite 1 und 310.277,61 € bei Visite 2 (Tab. 1; für weitere Details siehe Anhang 7; Tab. 2).

### Implementierungskosten und Kosten-Effektivitäts-Verhältnis-Analyse

Für die nachhaltige Integration der neuen Versorgungsform sind Koordinationsstellen an bundesweiten oder regionalen rheumatologischen Zentren erforderlich. Die jährlichen Personalkosten pro Zentrum, konservativ berechnet und auf Vollzeitäquivalente standardisiert, beliefen sich auf 198.993,60 € (Rheumatolog:innen: 107.608,80 €; RFA: 46.207,20 €; Sekretariatspersonal: 45.177,60 €). Basierend auf Daten aus Rheinland-Pfalz (3.039 Patient:innen), Niedersachsen (2.764 Patient:innen) und dem Saarland (773 Patient:innen), insgesamt 6.576 Patient:innen, die über 3 Jahre behandelt wurden, ergab sich ein jährlicher Durchschnitt von 2.192 Patient:innen und damit ein Zusatzkostenbetrag von 272,35 € pro Patient:in.

Insgesamt erzielte die neue Versorgungsform Kosteneinsparungen in Höhe von 3.838.845,07 € im Vergleich zur Kerndokumentation, davon 3.413.239,71 € bei Visite 1 und 425.605,36 € bei Visite 2. Dies entspricht jährlichen Einsparungen von 1.279.615,02 €. Selbst unter Einbeziehung der Kosten der Koordinationsstellen resultiert die neue Versorgungsform in erheblichen Nettoeinsparungen (Tab. 2).

Die Kosten-Effektivitäts-Verhältnis-Analyse zeigte, dass Rheuma-VOR ein dominantes Kosten-Effektivitäts-Profil aufweist und bei niedrigeren Gesamtkosten gleichwertige oder bessere Ergebnisse erzielt. Für Patient:innen mit vollständigen Daten betrugen die Kosten pro 0,1-Punkte(10 %)-Verbesserung der Lebensqualität zwischen Visite 1 und Visite 2 2.445,17 € in der Rheuma-VOR-Kohorte und 3.107,33 € in der Kerndokumentation, was eine überlegene Kosteneffizienz von Rheuma-VOR zeigt.

**Tab. 2:** Gesamtkostenübersicht bei Rheuma-VOR und der Kerndokumentation

	Rheuma-VOR in €	Kerndokumentation in €	Ersparnis/Mehrkosten in €
Medikamentenkosten V1	946.075,05	2.815.947,00	−1.869.871,95
Medikamentenkosten V2	1.345.276,00	1.508.682,27	−163.406,27
Krankenhausaufenthalte V1	978.131,75	2.077.948,37	−1.099.816,62
Krankenhausaufenthalte V2	519.999,38	573.662,99	−53.663,61
Rehabilitationsleistungen V1	167.575,30	225.619,94	−58.044,64
Rehabilitationsleistungen V2	224.308,21	122.566,08	+101.742,13
Produktivitätsverluste derzeit AU V1	1.298.006,06	1.228.399,80	+69.606,26
Produktivitätsverluste derzeit AU V2	447.100,56	737.033,55	−289.932,99
Produktivitätsverluste letzte 12 Monate AU V1	2.215.666,16	2.670.778,92	−455.112,76
Produktivitätsverluste letzte 12 Monate AU V2	1.614.529,80	1.634.874,42	−20.344,62
Implementierungskosten je Koordinationsstelley und Jahr	198.993,60	–	+198.993,60
*Gesamt V1*	5.605.454,32	9.018.694,03	−3.413.239,71
*Gesamt V2*	4.350.207,55	4.576.819,31	−226.611,76

Die Zeile Gesamt V2 enthält die Implementierungskosten der Koordinationsstelle in Höhe von 198.993,60 €. Ohne diese beträgt die Ersparnis zu Visite 2 −425.605,36 €. Die im Text ausgewiesenen Gesamteinsparungen sind vor Implementierungskosten angegeben.

Bei Visite 2 lagen für 463 Patient:innen (78,6 %) der Rheuma-VOR-Kohorte Angaben zur Häufigkeit rheumatologischer Facharztkontakte seit Visite 1 vor. Im Mittel wurden 3,48 Kontakte (SD 1,67) dokumentiert; der Median lag bei 4 Besuchen und entsprach damit der empfohlenen Regelversorgung [27]. Vergleichbare Daten waren für die Kerndokumentation nicht verfügbar.

## Diskussion

### Gesundheitsökonomische Grundlagen

Die gesundheitsökonomische Bewertung bekräftigt die in der Literatur bereits vielfach belegte Evidenz, dass entzündlich-rheumatische Erkrankungen mit erheblichen Kosten verbunden sind und dass eine Verbesserung der Versorgung und Behandlung sowohl zu reduzierten Ausgaben als auch zu einer höheren Lebensqualität führt [7–10].

Aus gesellschaftlicher Perspektive werden direkte und indirekte Kosten analysiert, um die ökonomische Belastung der Erkrankung („cost of illness“) darzustellen; Interventionen lassen sich mittels Kosteneffektivität, etwa über das inkrementelle Kosten-Effektivitäts-Verhältnis (ICER), vergleichen [6,28].

### Zentrale Studienergebnisse

Die Ergebnisse bestätigen die wachsende Evidenz zur Bedeutung einer frühzeitigen Diagnosestellung und Behandlung chronischer entzündlich-rheumatischer Erkrankungen für den Krankheitsverlauf und einer gesundheitsökonomischen Effizienz [3,16]. Eine rheumatologische Therapieinitiierung innerhalb von 3 Monaten nach Symptombeginn – wie von EULAR empfohlen – erhöht die Remissionswahrscheinlichkeit, senkt die Krankheitsaktivität und verhindert bzw. reduziert irreversible Schäden [3,4]. In der Praxis bestehen jedoch weiterhin erhebliche Verzögerungen: Die medianen Diagnoseintervalle betragen 4 Monate bei RA und 18 Monate bei axSpA [5], verursacht vor allem durch strukturelle Engpässe, insbesondere Fachärztemangel, sowie Defizite an den Schnittstellen der Versorgung [2].

Das Rheuma-VOR-Modell setzt genau hier an: Über sektorenübergreifende Koordinationsstellen mit strukturierter Triage und priorisierter Terminvergabe adressiert es genau diese Schnittstellenproblematik, verkürzt diagnostische Wartezeiten und ermöglicht die frühzeitige Einleitung leitliniengerechter Basistherapien. Dadurch sinkt nicht nur der Bedarf an kostenintensiven Eskalationstherapien, sondern führte auch zu einer schnelleren Kontrolle der Krankheitsaktivität, was wiederum weniger stationäre Aufenthalte und kürzere Arbeitsunfähigkeitszeiten bedingen könnte [11].

Die gesundheitsökonomische Evaluation des Rheuma-VOR-Modells belegt, dass die Implementierung strukturierter Koordinationsstellen bei entzündlich-rheumatischen Erkrankungen zu signifikanten Kosteneinsparungen und gleichwertigen, bzw. besseren, klinischen Ergebnissen im Vergleich zur Standardversorgung führt [11]. Über alle Ressourcenbereiche hinweg ergaben sich Gesamteinsparungen von 3,84 Mio. €, wobei der größte Anteil auf die Reduktion von Krankenhausaufenthalten und Arzneimittelausgaben entfiel. Besonders ausgeprägt war der Rückgang der Biologika-Verordnungen zu Beginn der Versorgung: In der Interventionsgruppe lag der Anteil der Patient:innen mit Biologika bei der ersten Visite bei 2,4 %, verglichen mit 11,8 % in der Kontrollgruppe, obgleich die Differenz wahrscheinlich unterschiedliche Krankheitsstadien zum Indexzeitpunkt reflektieren. Somit könnte man auch von dem monetären Effekt aufgrund der Verschiebung des Therapiestarts sprechen. Diese Befunde sind jedoch konsistent mit den Ergebnissen von Huscher et al. (2015), die aufzeigen, dass direkte Kosten – insbesondere für Arzneimittel und Krankenhausaufenthalte – einen erheblichen Anteil der Gesamtkosten entzündlich-rheumatischer Erkrankungen ausmachen und dass Verbesserungen in der Versorgung das Potenzial haben, diese Kosten zu senken [16].

Auf der Ebene der indirekten Kosten resultierten aus der verbesserten Arbeitsfähigkeit Einsparungen von rund 0,70 Mio. € über beide Erhebungszeitpunkte hinweg. Ein leichter Anstieg der Rehabilitationskosten bei der zweiten Visite könnte auf präventive Effekte hindeuten, die langfristig zur weiteren Kostenstabilisierung beitragen.

Die Implementierungskosten für die Koordinationsstellen beliefen sich auf durchschnittlich 272,35 € pro Patient:in. Selbst unter konservativen Annahmen generierte die Intervention einen deutlichen Nettovorteil. Nach Abzug der Implementierungskosten von 198.993,60 € verbleibt eine Nettoeinsparung von 3.639.851,47 €.

Damit adressiert Rheuma-VOR nicht nur klinische Versorgungsdefizite, sondern auch die finanzielle Nachhaltigkeit des Gesundheitssystems. Die Kosten-Effektivitäts-Verhältnis-Analyse (ICER) bestätigte ein dominantes Kosten-Effektivitäts-Profil: Rheuma-VOR erzielte eine vergleichbare oder höhere Verbesserung der gesundheitsbezogenen Lebensqualität zu geringeren Gesamtkosten.

Angesichts demografischer Entwicklungen und steigender Prävalenz chronischer Erkrankungen stellt Rheuma-VOR ein skalierbares, entlastendes Ergänzungsmodell dar, dessen bundesweite Einführung – selbst unter Einbeziehung der Implementierungskosten – zu Nettoeinsparungen in Millionenhöhe führen würde und somit klinischen Nutzen mit finanzieller Nachhaltigkeit verbindet [2].

Für eine flächendeckende Einführung sind jedoch strukturelle Rahmenbedingungen zu beachten: Der anhaltende Fachkräftemangel in der Rheumatologie, insbesondere in ländlichen Regionen, stellt eine wesentliche Limitation dar [2]. Eine reine Ausweitung des Modells ohne parallele Maßnahmen zur Fachkräftegewinnung und -bindung könnte das Potenzial der Intervention begrenzen. Ergänzende Strategien – wie die Einbindung telemedizinischer Angebote, die Delegation an rheumatologische Fachassistenzen sowie eine verbesserte hausärztliche Triage – erscheinen daher notwendig, um die Versorgungskapazitäten zu erhöhen [2].

### Methodische Limitationen und Stärken

Wie bei jedem Studientyp weist auch die vorliegende gesundheitsökonomische Evaluation methodische Stärken und Limitationen auf. Die Einbettung in das prospektive, multizentrische Rheuma-VOR-Projekt ermöglichte Analysen unter Alltagsbedingungen in einer großen, heterogenen Patient:innenpopulation und stärkte damit die externe Validität. Die fehlende Randomisierung birgt jedoch ein Restrisiko für Residualkonfounding, das durch Propensity-Score-Matching zwar reduziert, aber nicht vollständig ausgeschlossen werden konnte.

Eine zentrale methodische Herausforderung betrifft die Vergleichbarkeit des Indexzeitpunkts zwischen der Rheuma-VOR-Kohorte und der Kohorte der Kerndokumentation. Während die erste Visite in Rheuma-VOR definitionsgemäß den initialen rheumatologischen fachärztlichen Kontakt im Versorgungspfad abbildet, entspricht die erste in der Kerndokumentation erfasste Visite nicht zwingend dem ersten rheumatologischen Kontakt. Dadurch besteht die Möglichkeit, dass unterschiedliche Krankheitsstadien miteinander verglichen wurden. Auch wenn das Matching beobachtbare Kovariaten angleicht, kann eine strukturell unterschiedliche Definition des Indexzeitpunkts nicht vollständig kompensiert werden. Dies kann insbesondere die Interpretation therapiebezogener Endpunkte sowie der Arzneimittelkosten beeinflussen. Wesentlicher Kostenfaktor bei den Arzneimittelkosten sind Biologika, die aufgrund ihrer Preisstrukturen einen großen Einfluss auf die Gesamtkosten haben. Zum Zeitpunkt von Visite 2 sind keine signifikanten Unterschiede bei der Anzahl an Biologikaverordnungen erkennbar und somit könnten sich hier auch Effekte des Indexzeitraums auf die Kostenstrukturen zeigen.

Dennoch sind auch zu diesem Zeitpunkt beim Mittelwert der Kosten letztlich moderate Einspareffekte erkennbar. Ein ähnliches Muster zeigt sich bei den Krankenhauskosten. Summiert man die Einsparungen dieser beiden Kostenbereiche ausschließlich zu V2, zeigt sich eine vollständige Kompensation der Implementierungskosten der Koordinationsstellen. Selbst unter einem Szenario partiell differierender Krankheitsstadien ist aufgrund der Höhe der Differenzen von Einsparungen im langfristigen Zeitverlauf auszugehen, obgleich zu berücksichtigen ist, dass eine gegebenenfalls mögliche Umstellung von Biologika auf Biosimilars aufgrund des Studiendesigns nicht nachvollziehbar ist.

Weitere Herausforderungen ergaben sich aus der Nutzung von Registerdaten. Trotz umfassender Verfahren zur Datenharmonisierung und Plausibilitätsprüfung blieben Limitationen durch unvollständige oder inkonsistente Dokumentationen bestehen. Besonders betroffen waren Visitedaten (in der Kerndokumentation teilweise nur monatsgenau verfügbar) sowie Medikationsdaten mit fehlenden Enddaten oder unklarer zeitlicher Zuordnung. Vordefinierte Annahmen – beispielsweise die Interpretation fehlender Absetzangaben als fortlaufende Therapie – stärkten die Konsistenz der Auswertung, können jedoch systematische Verzerrungen verursachen. Hinzu kommt, dass in den verfügbaren Daten keine valide Differenzierung zwischen Biologika-Originalpräparaten und Biosimilars möglich war, sodass die Kostenkalkulation auf Basis der Referenzpräparate erfolgte. Da insbesondere Biologikatherapien einen wesentlichen Anteil an den Gesamtkosten ausmachen, könnten hierdurch Arzneimittelkosten potenziell überschätzt worden sein. Für die Monetarisierung der Krankenhausaufenthalte wurden außerdem literaturbasierte Tagessätze verwendet die sich in der aktuellen Versorgungspraxis verändert haben könnten. Bei der Berechnung der indirekten Kosten für Produktivitätsverluste wurde ein Friktionszeitraum von 90 Tagen angenommen, was eine konservative Annahme darstellt. Die in diesem Beitrag ausgewiesenen Gesamt- und Durchschnittswerte wurden aus den Einzelpositionen der zugrunde liegenden Auswertung berechnet.

Die große Stichprobengröße erhöht zwar die Generalisierbarkeit der Ergebnisse, kann jedoch Unterschiede in Dokumentations- oder Kodierpraktiken zwischen Zentren nicht vollständig kompensieren. Kausale Schlussfolgerungen sind aufgrund des beobachtenden Studiendesigns nicht möglich; beobachtete Effekte könnten zudem durch externe Faktoren, etwa regionale Versorgungsunterschiede, beeinflusst sein. Dies kann auch zu unterschiedlichen Fallzahlen in der Regelversorgung führen, die insbesondere in strukturschwachen Regionen eingeschlossen werden können. Ambulant-fachärztliche Kosten (EBM-Leistungen, GOÄ, Praxisbesuche) konnten in dem Modell außerdem nicht als eigene Kostenposition berücksichtigt werden.

Der Beobachtungszeitraum war auf zwölf Monate beschränkt, sodass langfristige Kosten- und Outcome-Effekte – beispielsweise durch die Vermeidung irreversibler struktureller Schäden – nicht erfasst werden konnten. Geplante Nachuntersuchungen sowie die perspektivische Einbindung von Krankenkassendaten sollen diese Limitation adressieren. Insgesamt erlaubt die Studie jedoch eine erste realitätsnahe Bewertung der Kosteneffektivität eines innovativen Versorgungsmodells und liefert relevante Evidenz für gesundheitspolitische Entscheidungen zur Optimierung der rheumatologischen Versorgung in Deutschland.

### Perspektive und Implikationen

Die vorliegenden Ergebnisse verdeutlichen, dass die frühzeitige Erkennung und strukturierte Versorgung entzündlich-rheumatischer Erkrankungen nicht nur klinische Vorteile, sondern auch signifikante gesundheitsökonomische Potenziale birgt. Das im Rahmen von Rheuma-VOR etablierte Koordinationsstellenmodell hat sich als effektiver Ansatz erwiesen, um diagnostische Verzögerungen zu reduzieren, den Zugang zu leitliniengerechter Therapie zu beschleunigen und gleichzeitig die Kostenbelastung für das Gesundheitssystem zu senken [11].

Die beobachteten Einsparungen bei Krankenhausaufenthalten, Arzneimittelausgaben und Produktivitätsverlusten unterstreichen, dass Investitionen in koordinierte Versorgungsmodelle nicht nur kostendeckend, sondern netto kostensparend wirken können.

Es ist jedoch zu berücksichtigen, dass auch die Einführung einer Triage nicht das grundsätzliche Problem des fachärztlichen rheumatologischen internistischen Mangels löst, sondern lediglich die begrenzten personellen und somit zeitlichen Ressourcen optimiert. Diese sind jedoch ebenfalls kapazitätsbegrenzt. Hierfür sind neben der Ausweitung der Weiterbildungsplätze auch innovative Delegations- und Substitutionsmodelle, die Einbindung digitaler Lösungen sowie eine engere Verzahnung zwischen haus- und fachärztlicher Versorgung erforderlich [2]. Rheuma-VOR kann dabei als struktureller Kern einer umfassenderen Versorgungsstrategie dienen, der durch regionale Anpassungen und sektorenübergreifende Kooperationen ergänzt wird.

Darüber hinaus legen die Ergebnisse nahe, dass eine frühzeitige Diagnosestellung langfristig zusätzliche positive Effekte entfalten dürfte, die über den Beobachtungszeitraum der Studie hinausgehen – etwa durch die Vermeidung irreversibler struktureller Schäden, die Reduktion chronischer Krankheitslast und die Erhöhung der Erwerbsfähigkeit [3]. Diese langfristigen Effekte könnten die Kosteneffizienz des Modells weiter steigern und gleichzeitig die Lebensqualität der Betroffenen nachhaltig verbessern.

Insgesamt belegen die Ergebnisse, dass eine strategische Investition in koordinierte, frühdiagnostische Versorgungskonzepte nicht nur die gesundheitlichen Outcomes der Patient:innen verbessert, sondern zugleich erhebliche volkswirtschaftliche Vorteile generieren kann [11]. Angesichts der prognostizierten Zunahme chronischer Erkrankungen in einer alternden Gesellschaft ist die zeitnahe Umsetzung solcher Modelle ein entscheidender Schritt, um die Leistungsfähigkeit und Aufrechterhaltung des Gesundheitssystems zu sichern [2].

### Schlussfolgerung

Zusammenfassend zeigt sich, dass die frühzeitige Erkennung und leitliniengerechte Behandlung entzündlich-rheumatischer Erkrankungen nicht nur das individuelle Krankheitsleiden reduziert, sondern auch substanzielle ökonomische Einsparungen ermöglicht. Das im Projekt Rheuma-VOR erprobte Versorgungsmodell mit zentralen Koordinationsstellen, strukturierter Triage und beschleunigter Terminvermittlung verkürzt die Zeit bis zur Diagnose und Therapieeinleitung, senkt den Bedarf an kostenintensiven Eskalationsbehandlungen, Krankenhausaufenthalten und längeren Arbeitsausfällen und erzielt vergleichbare Verbesserungen der Lebensqualität bei geringeren Gesamtkosten [11].

Für die Versorgungspraxis impliziert dies, dass eine flächendeckende Einführung solcher Koordinationsstellen einen wesentlichen Beitrag zur temporären Schließung bestehender Versorgungslücken leisten könnte, insbesondere in Regionen mit geringer Facharztdichte. Angesichts des bestehenden Rheumatolog:innenmangels erfordert eine nationale Implementierung jedoch zusätzliche strukturelle Maßnahmen und darüber hinaus zielführende Lösungsstrategien [2].

## Fazit für die Praxis

- Strukturelle Umsetzung: Einrichtung zentraler Koordinationsstellen an rheumatologischen Zentren mit klaren Überweisungswegen.
- Regionale und bedürfnisorientierte Anpassung: In ländlichen Gebieten Kombination mit mobilen Screening-Angeboten oder telemedizinischer Anbindung.
- Fachkräftemangel aktiv begegnen – z. B. durch Delegation an speziell geschulte rheumatologische Fachassistenzen, Ausbau der Weiterbildungsplätze und Nutzung digitaler Lösungen.
- Hausärztliche Triage stärken – gezielte Schulung und klare Zuweisungskriterien, um Verdachtsfälle schneller in die Fachversorgung zu bringen.

## Supplementary Information

Anhang 1 bis 7

## Data Availability

Die im Rahmen der Studie erhobenen Originaldaten sind auf begründete Anfrage erhältlich.
