# Supplementary material for: Rheuma-VOR—Health economic evaluation of early rheumatological diagnosis: Summary of the G-BA report
Source: Z Rheumatol. 2026 Sep 18;85(8):595–607. [Article in German] doi: 10.1007/s00393-026-01858-6 (PMC13649902; doi:10.1007/s00393-026-01858-6)
Supplement: Supplementary file 1 [file 393_2026_1858_MOESM1_ESM.docx]

**Anhang:**

**Rheuma-VOR – gesundheitsökonomische Bewertung einer frühzeitigen rheumatologischen Diagnostik. Zusammenfassung des G-BA-Berichtes**

Anhang 1: Bei Diagnose verschriebene Biologika

|  | **Rheuma-VOR** | | | | **DRFZ** | | | |
| --- | --- | --- | --- | --- | --- | --- | --- | --- |
| **Hauptdiagnose** | **Vis. 1 ges.** | **Vis. 1 von 2** | **Vis. 2** | **Verschreibung erstmals im Studienzeitraum** | **Vis. 1 ges.** | **Vis. 1, wenn 2 Vis. vorhanden** | **Vis. 2** | **Verschreibung erstmals im Studienzeitraum** |
| Rheumatoide Arthritis | 22 | 5 | 38 | 58 | 99 | 52 | 79 | 115 |
| Psoriasis Arthritis | 5 | 10 | 31 | 32 | 41 | 28 | 43 | 54 |
| Spondylopathien | 7 | 8 | 17 | 22 | 25 | 21 | 31 | 34 |
| **Summe** | 34 | 23 | 86 | 112 | 165 | 101 | 153 | 203 |

Anhang 1 folgt der Auswertung nach Hauptdiagnosen und ordnet jeder Patient:in genau einer Zeile zu, Anhang 2 folgt der Auswertung nach Wirkstoffen und weist die Verordnungen je Substanz aus. Zu Visite 1 stimmt die Summenzeile mit Tab. 1 überein (Rheuma-VOR 34, Kerndokumentation 165). Für die Kohorte der Kerndokumentation weichen beide Auswertungen zu Visite 2 voneinander ab; der in Tab. 1 ausgewiesene Wert beruht dort auf der Wirkstoffaufstellung (49 + 45 = 94). Für die Rheuma-VOR-Kohorte stimmen beide Wege auch zu Visite 2 überein (9 + 77 = 86). Die Ursache der Abweichung ließ sich anhand der vorliegenden Unterlagen nicht abschließend klären.

Anhang 2: Verschriebene Biologika an n (%) Patienten bei Vis. 1 bzw. 2

|  | **Rheuma-VOR** | | **DRFZ** | |
| --- | --- | --- | --- | --- |
| Biologika, n (%) | Vis. 1 & 2 | Nur Vis. 2 | Vis.1 & 2 | Nur Vis. 2 |
| Abatacept |  | 11 (1,9) | 2 (0,3) | 3 (0,5) |
| Adalimumab | 3 (0,5) | 32 (5,4) | 17 (2,9) | 16 (2,7) |
| Anakinra |  |  | 2 (0,3) |  |
| Certolizumab | 2 (0,3) | 3 (0,5) | 6 (1,0) | 7 (1,2) |
| Etanercept | 3 (0,5) | 24 (4,1) | 16 (2,7) | 9 (1,5) |
| Golimumab | 1 (0,2) | 2 (0,3) | 4 (0,7) | 4 (0,7) |
| Infliximab |  | 1 (0,2) |  | 1 (0,2) |
| Rituximab |  | 3 (0,5) |  |  |
| Tocilizumab |  | 1 (0,2) | 2 (0,3) | 5 (0,8) |
| **Gesamt** | **9** | **77** | **49** | **45** |
| **Vergleich Rheuma-VOR gegenüber Kerndokumentation zu Visite 2. Signifikanz** | **p = 0,743** | | | |

Anhang 3: Verschriebene Medikamente n (%) Vis. 1 gesamt

| **Verschriebene Medikamente, n (%) bei Vis. 1** | **Rheuma-VOR** | **DRFZ** | **Signifikanz** |
| --- | --- | --- | --- |
| Methotrexat oral | 188 (13,5) | 291 (20,9) | p < 0,001 |
| Methotrexat parenteral | 576 (41,3) | 390 (28,0) | p < 0,001 |
| Kortikoide | 616 (44,2) | 559 (40,1) | p = 0,029 |
| Sulfasalazin | 71 (5,1) | 126 (9,0) | p < 0,001 |
| Hydroxychloroquin | 36 (2,6) | 65 (4,7) | p = 0,003 |
| Leflunomid | 41 (2,9) | 75 (5,4) | p = 0,001 |
| Auranofin | 1 (0,1) |  |  |
| Chloroquin |  | 7 (0,5) |  |
| Azathioprin | 1 (0,1) | 4 (0,3) |  |
| **Gesamt** | **1.530** | **1.517** |  |

Anhang 4: Verschriebene Medikamente n (%) Vis. 1 & 2 und nur Vis. 2 gesamt

|  | **Rheuma-VOR** | | **DRFZ** | | **Signifikanz** |
| --- | --- | --- | --- | --- | --- |
| Medikamente, n (%) | Vis. 1 & 2 | Nur Vis. 2 | Vis. 1 & 2 | Nur Vis. 2 | Vis. 2 |
| Methotrexat oral | 35 (5,9) | 24 (4,1) | 117 (19,9) | 45 (7,6) | p < 0,001 |
| Methotrexat parenteral | 171 (29,0) | 50 (8,5) | 167 (28,4) | 50 (8,5) | p = 0,810 |
| Kortikoide | 93 (15,8) | 29 (4,9) | 219 (37,2) | 34 (5,8) | p < 0,001 |
| Sulfasalazin | 13 (2,2) | 21 (3,6) | 50 (8,5) | 12 (2,0) | p = 0,003 |
| Azathioprin |  | 4 (0,7) | 3 (0,5) |  |  |
| Hydroxychloroquin | 6 (1) | 11 (1,9) | 28 (4,8) | 10 (1,7) | p = 0,004 |
| Leflunomid | 4 (0,7) | 30 (5,2) | 30 (5,1) | 29 (4,9) | p = 0,007 |
| Chloroquin |  | 1 (0,2) | 4 (0,7) | 3 (0,5) | p = 0,033 |
| **Gesamt** | **322** | **170** | **618** | **183** |  |

Anhang 5: Krankenhausaufenthalte aufgrund rheumatischer Erkrankungen

|  | **Stationärer KH-Aufenthalt in letzten 12 Monaten n (%)** | **Dauer KH-Aufenthalt in Tagen, MD (SD) Median** |
| --- | --- | --- |
| **Rheuma-VOR** |  |  |
| Vis. 1 gesamt | 101 (7,2) | 14,32 (29,64) 10 |
| Nur Vis. 1 | 52 (6,5) | 17,64 (39,07) 10 |
| Vis. 1 von 2 | 49 (8,3) | 10,85 (14,19) 9,5 |
| Nur Vis. 2 | 55 (9,3) | 13,98 (7,57) 14 |
| **DRFZ** |  |  |
| Vis. 1 gesamt | 219 (15,7) | 14,03 (18,60) 10 |
| Nur Vis. 1 | 125 (15,5) | 12,94 (12,34) 10 |
| Vis. 1 von 2 | 94 (16,0) | 15,50 (24,69) 10 |
| Nur Vis. 2 | 75 (12,7) | 11,31 (6,69) 10 |
| **Signifikanz** |  |  |
| Vis. 1 gesamt | p < 0,001 | p = 0,085 |
| Nur Vis. 1 | p < 0,001 | p = 0,533 |
| Vis. 1 von 2 | p < 0,001 | p = 0,043 |
| Nur Vis. 2 | p = 0,002 | p = 0,019 |

Anhang 6: Rehabilitationsleistungen

|  | **Reha in letzten 12 Monaten, n (%)** | | | |  |
| --- | --- | --- | --- | --- | --- |
|  | **gesamt** | **ambulant** | **stationär** | **ambulant & stationär** | **Dauer stationäre Reha in Wochen, MW (SD) Median** |
| **Rheuma-VOR** |  |  |  |  |  |
| Vis. 1 gesamt | 73 (5,2) | 29 (2,1) | 41 (2,9) | 3 (0,2) | 4,20 (2,76) 3 |
| Nur Vis. 1 | 38 (4,7) | 19 (2,5) | 18 (2,2) | 1 (0,1) | 4,10 (2,97) 3 |
| Vis. 1 von 2 | 35 (5,9) | 10 (1,7) | 23 (3,9) | 2 (0,3) | 4,30 (2,46) 3 |
| Nur Vis. 2 | 90 (15,2) | 23 (3,9) | 65 (11,0) | 2 (0,3) | 4,56 (4,40) 3 |
| **DRFZ** |  |  |  |  |  |
| Vis. 1 gesamt | 120 (8,6) | 49 (3,5) | 68 (4,9) | 3 (0,2) | 3,44 (1,35) 3 |
| Nur Vis. 1 | 70 (8,6) | 30 (3,7) | 39 (4,8) | 1 (0,1) | 3,69 (1,61) 3 |
| Vis. 1 von 2 | 50 (8,4) | 19 (3,2) | 29 (4,9) | 2 (0,3) | 3,15 (0,91) 3 |
| Nur Vis. 2 | 65 (11,0) | 20 (3,4) | 43 (7,3) | 2 (0,3) | 3,45 (1,89) 3 |
| **Signifikanz** |  |  |  |  |  |
| Vis. 1 gesamt | p < 0,001 |  |  |  | p = 0,288 |
| Nur Vis. 1 | p < 0,001 |  |  |  | p = 0,720 |
| Vis. 1 von 2 | p < 0,001 |  |  |  | p = 0,055 |
| Nur Vis. 2 | p < 0,001 |  |  |  | p = 0,027 |

Anhang 7: Produktivitätsverluste aufgrund der rheumatischen Erkrankung

|  | **Derzeit krankgeschrieben, n (%)** | **In letzten 12 Monaten krankgeschrieben n (%)** | **Anzahl krankgeschr. Tage, MW (SD) Median** |
| --- | --- | --- | --- |
| **Rheuma-VOR** |  |  |  |
| Vis. 1 gesamt | 215 (15,4) | 367 (26,3) | 38,15 (64,96) 15 |
| Nur Vis. 1 | 119 (14,8) | 214 (26,6) | 41,93 (71,59) 15 |
| Vis. 1 von 2 | 96 (16,3) | 153 (26,0) | 32,86 (54,16) 16,5 |
| Nur Vis. 2 | 36 (6,1) | 130 (22,1) | 78,48 (123,39) 20 |
| **DRFZ** |  |  |  |
| Vis. 1 gesamt | 155 (11,1) | 337 (24,2) | 50,08 (77,15) 21 |
| Nur Vis. 1 | 84 (10,8) | 194 (24,1) | 50,51 (79,28) 20 |
| Vis. 1 von 2 | 71 (12,1) | 143 (24,3) | 59,52 (74,52) 25 |
| Nur Vis. 2 | 55 (9,3) | 122 (20,7) | 84,68 (117,02) 24 |
| **Signifikanz** | Derzeit krankgeschrieben, n (%) | In letzten 12 Monaten krankgeschrieben n (%) | Anzahl krankgeschr. Tage, MW (SD) Median |
| Vis. 1 gesamt | p = 0,954 | p < 0,001 | p = 0,023 |
| Nur Vis. 1 | p = 0,702 | p < 0,001 | p = 0,332 |
| Vis. 1 von 2 | p = 0,731 | p < 0,001 | p = 0,017 |
| Nur Vis. 2 | p < 0,001 | p < 0,001 | p = 0,446 |
| MWU | 167814,000 | 135436,000 | 11524,500 |
| Wilcoxon-W | 298119,000 | 362237,000 | 27277,500 |
| Z | -1,080 | -3,588 | -1,674 |
| Asymptotische Sign. (2-seitig) | ,280 | ,000 | ,094 |
